# The reconfiguration pattern of individual brain metabolic connectome for Parkinson's disease identification

**DOI:** 10.1002/mco2.305

**Published:** 2023-06-27

**Authors:** Weikai Li, Yongxiang Tang, Liling Peng, Zhengxia Wang, Shuo Hu, Xin Gao

**Affiliations:** ^1^ College of Mathematics and Statistics Chongqing Jiaotong University Chongqing China; ^2^ Department of Nuclear Medicine (PET Center) XiangYa Hospital Changsha Hunan China; ^3^ Department of PET/MR Shanghai Universal Medical Imaging Diagnostic Center Shanghai China; ^4^ MIIT Key Laboratory of Pattern Analysis and Machine Intelligence Nanjing University of Aeronautics and Astronautics Nanjing China; ^5^ School of Computer Science and Cyberspace Security Hainan University Hainan China; ^6^ Key Laboratory of Biological Nanotechnology of National Health Commission XiangYa Hospital Central South University Changsha Hunan China

**Keywords:** FDG‐PET, JS divergence, metabolic brain network, MK‐SVM, Parkinson's disease

## Abstract

^18^F‐Fluorodeoxyglucose positron emission tomography (^18^F‐FDG PET) is widely employed to reveal metabolic abnormalities linked to Parkinson's disease (PD) at a systemic level. However, the individual metabolic connectome details with PD based on ^18^F‐FDG PET remain largely unknown. To alleviate this issue, we derived a novel brain network estimation method for individual metabolic connectome, that is, Jensen‐Shannon Divergence Similarity Estimation (JSSE). Further, intergroup difference between the individual's metabolic brain network and its global/local graph metrics was analyzed to investigate the metabolic connectome's alterations. To further improve the PD diagnosis performance, multiple kernel support vector machine (MKSVM) is conducted for identifying PD from normal control (NC), which combines both topological metrics and connection. Resultantly, PD individuals showed higher nodal topological properties (including assortativity, modularity score, and characteristic path length) than NC individuals, whereas global efficiency and synchronization were lower. Moreover, 45 most significant connections were affected. Further, consensus connections in occipital, parietal, and frontal regions were decrease in PD while increase in subcortical, temporal, and prefrontal regions. The abnormal metabolic network measurements depicted an ideal classification in identifying PD of NC with an accuracy up to 91.84%. The JSSE method identified the individual‐level metabolic connectome of ^18^F‐FDG PET, providing more dimensional and systematic mechanism insights for PD.

## Graphical abstract

We derived a novel brain network estimation method for the individual metabolic connectome, that is, Jensen‐Shannon Divergence Similarity Estimation method. Specifically, the JS divergence was utilized for measuring statistical association of resemblance of cerebral glucose metabolism between two regions. Further, the altered pattern of the connections, global, and nodal graph metrics in PD are discovered. Finally, to diagnose the PD, the MK‐SVM was implemented for combining the information from the connections, global, and nodal graph metrics.

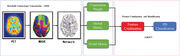

## 1 INTRODUCTION

With an estimated current global total of 6.2 million people affected, Parkinson's disease (PD) is second most prevalent neuro‐degenerative movement disorder. Its prevalence is expected to increase significantly in coming decades.^1^ Unfortunately, so far, diagnosis and assessment of severity of PD relies heavily on clinical examination and follow‐up. This disease is typically distinguished by motor indications, including tremor, rigidity, akinesia or bradykinesia, and nonmotor indications like psychiatric symptoms and cognitive impairment.^2^ Motor indications related to PD are classically associated with losing dopamine‐producing neurons in substantia nigra.^3^, ^4^, ^5^ However, PD pathology often involves neurotransmitters other than dopamine and is distributed among many other cortical and subcortical brain regions participating in neural networks,^6^, ^7^, ^8^, ^9^, ^10^ making the clinical diagnosis and differentiating of early PD stages challengeable. The error rates of PD diagnosis at clinical level may be 24%, even in specialized centers.^11^, ^12^

As one of effective functional neuroimaging techniques, ^18^F‐FDG PET (positron emission tomography with ^18^F‐fluorodeoxyglucose) measures metabolic abnormalities in PD at a systemic level.^13^, ^14^ Cerebral glucose metabolism, which serves as the main energy source for activity of neuron, is strongly linked to local neural function, integrity, as well as density. Previous studies have provided significant insight into the neurobiological basis of PD, the association of distinct brain regions with linked clinical profiles.^15^, ^16^ PD‐related pattern (PDRP) from cerebral metabolism has been identified since early days of ^18^F‐FDG PET imaging and now deemed PD biomarker in trials conducted at clinical level.^17^, ^18^, ^19^, ^20^, ^21^ As per standard PD criteria of clinical diagnosis,^22^, ^23^ neuroimaging of normal presynaptic dopamine transporter (DAT) is used to rule out PD. DAT imaging, however, has limited assessment in distinguishing PD from atypical Parkinsonism due to its limited specificity and whole‐brain system‐level analysis.^24^ In particular, several ^18^F‐FDG PET‐based machine learning approaches have successfully promoted that the accurate use of metabolic PET imaging for PD^25^, ^26^ and classified PD from normal controls (NCs), which has achieved an empirical .success.^27^, ^28^, ^29^ However, such methods naturally fail to consider the metabolic linkage of between‐regions, possibly losing important evidence related to individual variations of metabolic topology or network, and tended to be sensitive to the scanning parameters. Naturally, this leads to instability of the biomarker, as the connectome tends to be more robust for identification as a second‐order statistic.^30^

Intuitively, the brain metabolic network detected in PD can help for distinguishing the disease from NCs and offering extra poof in supporting parkinsonian pathophysiological processes.^31^ Individual metabolic network estimation can be clinically meaningful. Thus, an obvious goal is to reveal the robust brain metabolic network for PD analysis. Recently, several PET‐based strategies are suggested to study metabolic systems.^30^ Many of these metabolic connectome investigations, however, only concentrate on the group level, which inevitably leads to the loss of significant individual‐level information. To alleviate this issue, inspired by some recent individual morphological connectome studies using the distribution divergence to construct the individual network based on gray matter.^32^, ^33^ We have developed a unique methodology for generating individual‐level metabolic brain networks, known as Jensen‐Shannon Divergence Similarity Estimation (JSSE). It is noteworthy that several machine learning methods^34^, ^35^, ^36^ based on brain networks are suggested for analyzing PD^37^ and related neuro‐diseases, which yield notable results. Almost all existing approaches only focus on the connection weight pattern, falling in discover more biologically meaningful connectome information (e.g., topology information). Consequently, inspired by the multiview learning trick in the machine learning field,^38^ this study further simultaneously utilized the data of connection value, global graph measurement, and nodal graph measurement/metrics to accurately identify PD.

Our primary objectives were to investigate the changes in the individual brain metabolic connectome, specifically its global graph metrics, nodal graph metrics, and connections. Additionally, it was aimed to accurately diagnose PD patients in comparison to NCs, by utilizing the multikernel trick. We accomplished this by (i) identifying the greatest distinctive nodal characteristics of predominant and brain connectome brain portions in PDs, (ii) achieving precise and automated categorization of PD sufferers and NCs, and (iii) analyzing altered patterns in brain network. We present an individual‐level metabolic network construct approach for ^18^F‐FDG PET imaging and JSSE approach can depict individual pathophysiology details of PD and downstream clinical individual diagnosis. The results of disruption nodes and connections of metabolic network can further highlight individual neurophysiological details and the metabolic perturbations basal ganglia (BG) and other cortical connectivity that underlie the cardinal motor other symptomatic features of PD.

## 2 RESULTS

### 2.1 Global graph metrics of metabolic brain connectome

We reported global graph metrics of NC and PD in Table 1. Global graph metrics are estimated by Gretna.^39^ As we can observer, *A*_r_, *Q*, *H*_r_, *E*_local_,$\;C_{p}$, γ, λ, and *L*_p_ increased, whereas *E*_global_, σ, and *S*_r_ reduced in PD groups. Statistical analysis depicted that $A_{r},\;Q,\;\lambda ,$ and *L*_p_ of PD patients were substantially higher compared with those in NC group, illustrating the significantly modularity change in the PD patients. Besides, the *S*_r_ and *E*_global_ were lower in PD groups than NC groups (*p* less than 0.05), illustrating that the personalized abnormity of metabolic networks and the significantly information transportation change in the PD patients. All details of the graph measurements are given in the Supplementary Materials.

**TABLE 1:** Metabolic brain connectome with global graph metrics.

Global graph metrics	PD (mean ±SD)	NC (mean ±SD)	*p* Value
*A*_r_^*^	0.1502±0.02	0.1432±0.02	0.0469
$Q^{*}$	10.8503±1.25	10.4082±0.93	0.0419
Hr	0.0080±0.02	0.0068±0.01	0.8175
$E_{\mathrm{global}}^{*}$	0.2231±0.01	0.2274±0.00	0.0391
*E*_local_	0.3351±0.01	0.3339±0.01	0.4824
*C*_p_	0.2881±0.01	0.2862±0.01	0.1962
γ	0.6408±0.05	0.6261±0.04	0.1491
$\lambda^{*}$	0.5275±0.01	0.5210±0.01	0.0345
σ	0.5401±0.04	0.543±0.04	0.5183
$L_{p}^{*}$	1.0529±0.05	1.0374±0.04	0.0426
*S*_r_^*^	−0.6865±0.89	−0.1746±0.28	0.0007

*A*_r_, assortativity; *L*_p_, characteristic path length; Hr, hierarchy; NC, normal control; PD, Parkinson's disease; *Q*, modularity score; *S*_r_, synchronization; *γ*, normalized clustering coefficient; *E*_local_, local efficiency; *C*_p_, clustering coefficient; *λ*, normalized characteristic path length; *E*_global_, global efficiency; *σ*, small‐world.

*p‐value < 0.05.

### 2.2 Degree analysis for metabolic brain connectome

**FIGURE 1:**
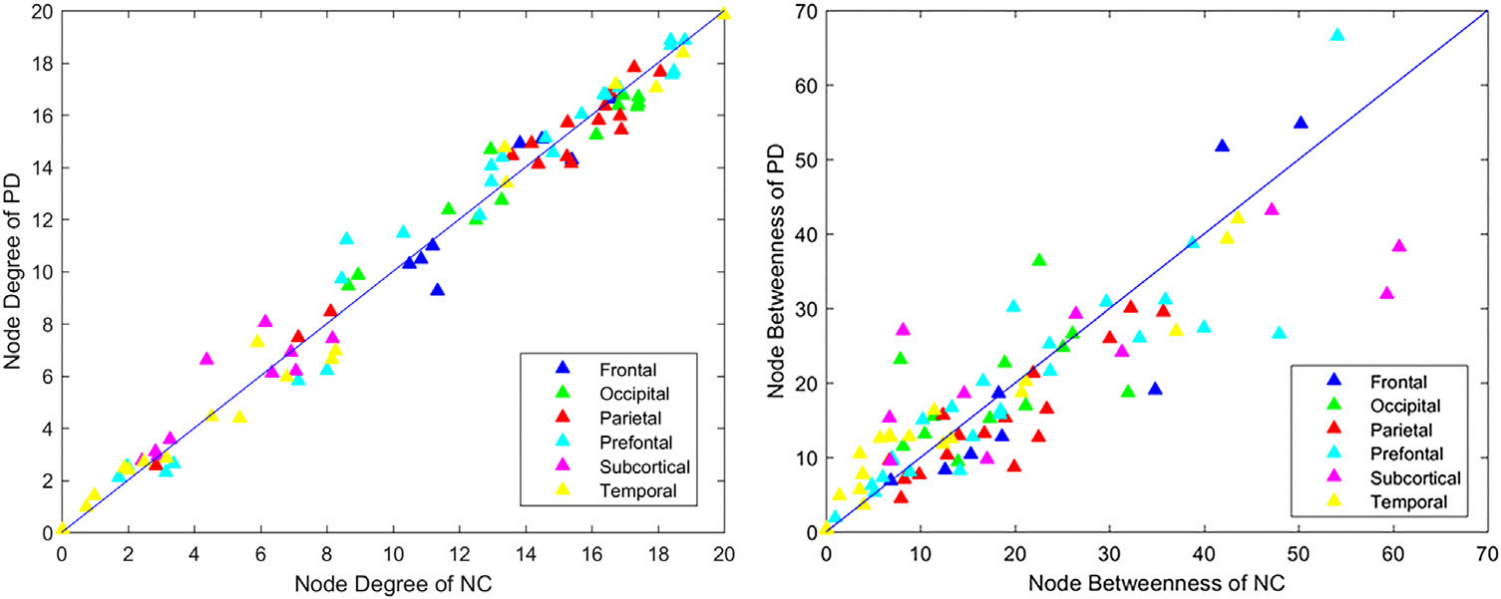
Distribution of degree (left) and betweenness (right) for NC and PD groups. Degree for parietal and frontal areas tends to be decreased in PD while tending to be increased in the prefrontal and subcortical regions. The betweenness in frontal and parietal regions tends to be decreased in PD while tending to be increased in the temporal and subcortical regions.

We visualized all node's mean degree of each node in PD and NC groups for investigating degree spreading of assessed metabolic brain connectome. Degree is a fundamental measure of node interconnectivity and reflects critical network evolution characteristics. Specifically, degree is stated as number of edges directly linked to a node. Greater a node's degree, more connections it has, and the more pivotal its role in the network. As exhibited in Figure 1, degree in parietal and frontal regions tends to be decreased for PD whereas tending to be increased in the prefrontal and subcortical regions. Specifically, the 19 important nodes with average degree in NC and PD groups are listed in the supplementary files (Table S1). The nodes having degree of standard deviations larger than mean of degree of every node were recognized as degree hub nodes.^40^ By comparing the hub nodes for two groups in similar modal system, it was obvious found that many were overlapped. Furthermore, it is imperative to note that many hub nodes existed that linked to various groups. For instance, in a metabolic network based on PET, the PCUN.L, SOG.R, and IOG.R only appeared in NCs hub nodes, whereas the MTG.R and PCUN.R only appeared in the PD group as the hub node, which can be linked to the nonmotor indications of PD. For the convenient presentation, ROI is represented by its abbreviation, which is defined in the Supplementary Material (Table S3). Moreover, L represents left, and R represents right. These findings underline that our individual‐level network construction methodology may detect more individual variation in the metabolic network organization, thus capturing more idiosyncratic or individual details (45 important connections).

### 2.3 Between analysis the metabolic brain connectome

Similarly, we visualized mean betweenness of NC and PD groups as shown in Figure 1 to examine betweenness spreading of assessed metabolic brain connectome. Betweenness is defined from the perspective of information flow and characterizes node importance in facilitating exchange of data in network. Specifically, betweenness is stated as number of path passing through this node. The result illustrates that betweenness in parietal and frontal regions tends to be decreased in PD while tending to be increased in subcortical and temporal areas. Fifteen major nodes of the average betweenness for NC and PD groups are given in the supplementary files (Table S2).

By comparing betweenness hub nodes between NC group and PD group in same modal system, many specialized hub nodes correspond to various groups. INS.R/L, ORBsup.L, SMG.R/L, ORBmid.R/L, and PCUN.R only appeared in hub nodes of NCs, while the IOG.L, SFGmed.R, and ACG.L only appeared in the PD group as the hub node. It also directly supports that PD pathology often involves neurotransmitters other than dopamine and is distributed across many other cortical and subcortical brain areas involved in neural networks.

### 2.4 Classification results

We utilized various quantitative indicators, such as area under curve (AUC), specificity, sensitivity, and accuracy to assess classification of information combination methodologies and suggested JSSE. More details can be found in the Supplemental Materials.

**FIGURE 2:**
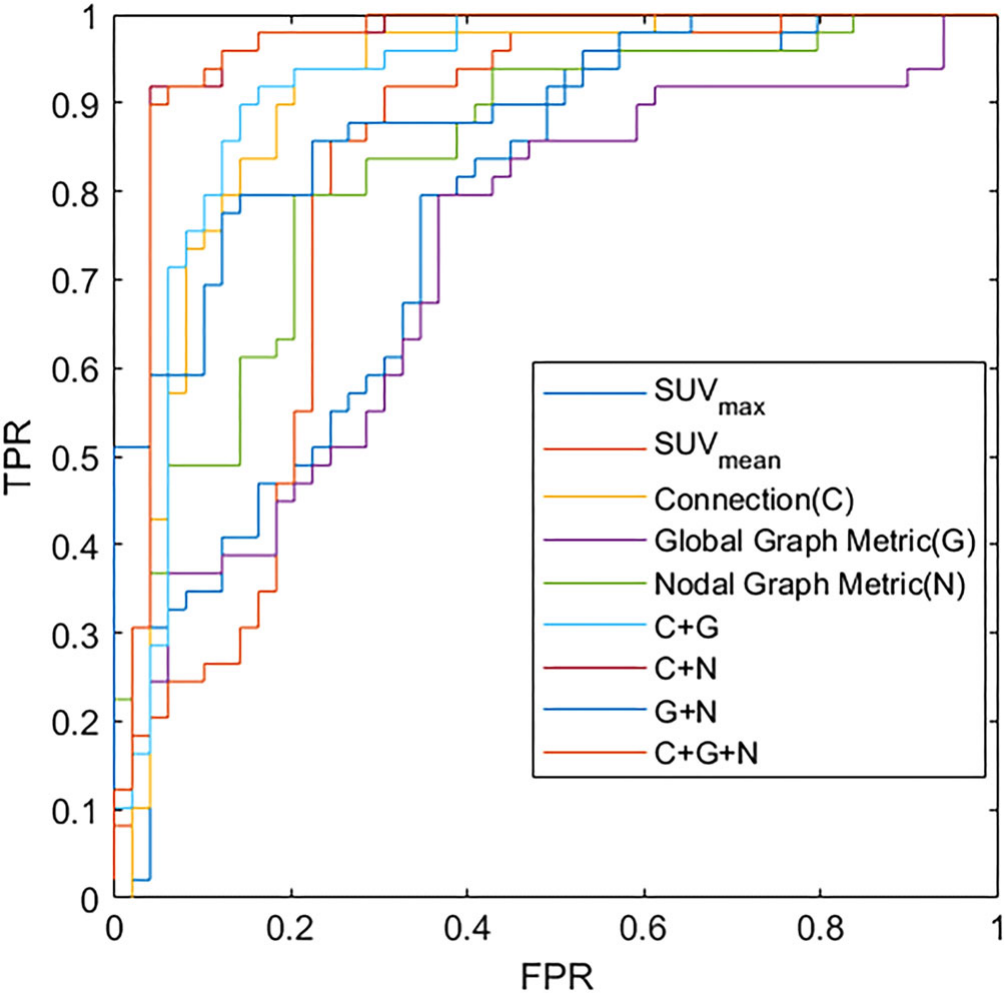
The ROC results of different methods. TPR, true positive rate; FPR, false positive rate.

We also reported single kernel support vector machine (SVM) classification outcome on the basis of nodal metrics, global metrics, and connection to validate information combination trick. In addition, the results of SUV_mean_ and SUV_max_ (mean and maximum standardized uptake value of each ROI) are also provided, in which the two values of each ROI are conduct as input to train classifiers. Findings are presented in Table 2, and ROC curve is depicted in Figure 2. Performance of the combined information methods is better than that of the single kernel methodologies, which supports logic of suggested approach. C+G+N methodology, which combines information of nodal metrics, global metrics, and connection, accomplished best outcomes in every four readings, elaborating its efficiency. Moreover, DeLong's nonparametric statistically important experiment showed that suggested C+G+N methodologies are substantially better than nodal, global, and connection methods with a 95% confidence interval (CI), having *p* values of 0.0482, $4\times 10^{-6}$, and 0.0115, respectively These results indicate that data combination strategy can efficiently enhance the classification outcome, and the ROC analysis for the mixed NC/PD group once more showed that it had excellent predictive performance.

**TABLE 2:** Various methods linked to the classification performance.

Method	Accuracy (%)	Sensitivity (%)	Specificity (%)	AUC
SUV_max_	78.57	85.71	71.42	0.7601
SUV_mean_	72.44	79.59	65.36	0.8113
Connection (C)	87.76	79.59	85.71	0.9058
Global (G)	65.31	63.27	67.35	0.7242
Nodal (N)	76.53	81.63	71.43	0.8354
C+G	87.76	89.80	85.71	0.9171
C+N	90.82	93.88	87.76	0.9566
G+N	81.63	77.55	85.71	0.8879
C+G+N	91.84	93.88	89.90	0.9571

*Note*: C+G+N methodologies are superior to nodal, global, and connection with 95% confidence interval having *p* values of 0.0115, $4\times 10^{-6}$, and 0.0482, respectively. AUC, area under curve.

### 2.5 Consensus significant metabolic connections

**FIGURE 3:**
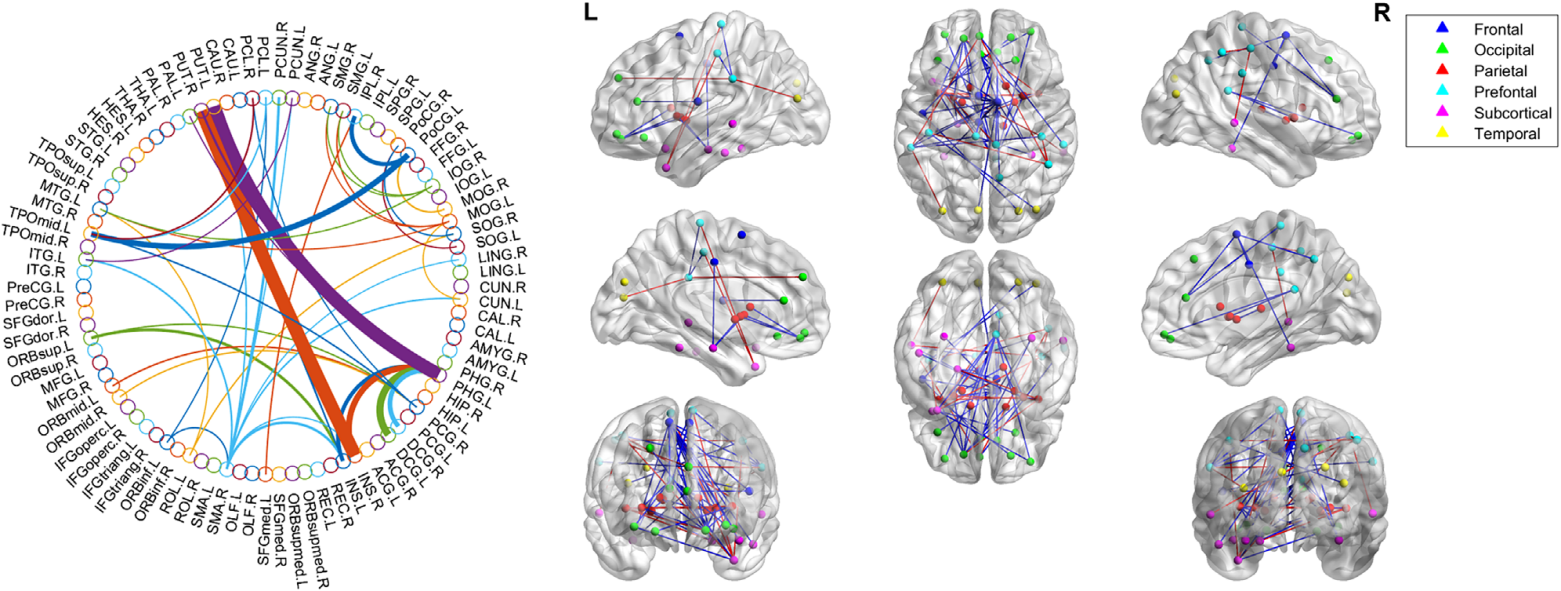
The most consensus connections. They were mapped on International Consortium for Brain Mapping (ICBM) 152 template using the BrainNet Viewer software package http://nitrc.org/projects/bnv/ and circularGraph, shared by Paul Kassebaumb http://www.mathworks.com/matlabcentral/fileexchange/48576‐circulargraph). (Left) The arc thickness reflects the distinguishing potential of the edge, which is inversely related to projected *p* values. L, left, R, right. (Right) Connectivity matrices of completely connected PD networks in comparison to NCs are depicted. 45 important connections were kept, red and green lines representing connection weights that are increased and decreased for PDs, correspondingly.

As previously revealed, we chose consensus connections with *p*‐ value of <0.05 for every loop. Since chosen connections in every inner cross‐validation loop may vary, we measured every chosen aspect in the total training procedure, i.e., the consensus connections. In total, we chose 45 consensus connections (as shown in Figure 3) with a *p* value of <0.05 in the inner validation loop. The majority of these consensus connections in frontal, prefrontal, and occipital regions exhibited decreased activity in PD patients, while those in the parietal and subcortical regions showed increased activity. The degree of the consensus connection shows that our proposed individual network method captures more than twice as many changes in SMA.R, PHG.L, INS.L/R, PCG.R, MOG.L, PUT.L/R, TPOmid.L, IOG.R, PoCG.L, and MTG.L that are activity in PD. Moreover, the novel application of the JSSE method discovered a large number of abnormal metabolic activity findings in the PCL.R, ITG.L, SOG.R, PoCG.R, IPL.R, SMG.L, SMG.R, PCUN.L, PCL.L, ORBsup.L, ORBinf.L, ROL.L, and CUN.L, this is in comparison with the group‐level analysis, which sheds new light on the network abnormality underlying the individual neurophysiological mechanisms of multitude of motor/nonmotor features from NC to PD.

## 3 DISCUSSION

Herein, we have introduced innovative analytical methodology for creating individual‐level metabolic brain systems using ^18^F‐FDG PET imaging and graph theory metrics. Our aim was to explore the variations of PD's metabolic connectome. Outcome of this study reveal compensatory mechanisms underlying PD. Furthermore, suggested classification methodology underscores ability of connectome‐based metrics in identifying PD. Developed classification methodology indicates capacity of connectome‐based metrics for identifying PD. Although ^18^F‐FDG PET based on metabolic PDRP and group‐level network methods has been shown to achieve a great success for PD analysis, such approaches cannot identify individual neurophysiological details and the metabolic perturbations BG and cortical linkage that cause cardinal motor aspects of PD. Ascendancy of our distinctive JSSE method may allow individual neurophysiological mechanisms of a multitude of nonmotor features.

The PDRP was recognized by Eidelberg et al.^41^, ^42^ for the time. Several studies have done PD diagnosis by using metabolic PDRP markers.^13^, ^43^ Metabolic system has high reproducibility and is selective for PD, which can help distinguish PD from NC.^44^ During group‐level analyses, PDRP pathophysiologic and clinical linkages are widely studied,^13^ which has potentially sacrificed or obscured salient individual differences within a group. Specifically, we used the JSSE to directly estimate the symmetric metabolic network based on the ^18^F‐FDG PET. Our individual metabolic JSSE system study revealed deviations of connection value, nodal graph measurement, and global graph measurement that were powerfully predictive of PD from NC. Our findings of decreased connectivity patterns in occipital, parietal, and frontal areas and increased in prefrontal, temporal, and subcortical regions, recapitulated the outcomes of earlier ^18^F‐FDG PET group‐based differences.^13^, ^15^, ^31^, ^45^ Our study has demonstrated that using JSSE to analyze ^18^F‐FDG PET data is promising methodology for uncovering an individual's metabolic connectivity networks and gaining new perceptions of nature of metabolic instability for PD clinical characteristics. From relative entropy evaluation, we have shown that this approach offers a novel perspective that could be useful in future studies of PD.

Neuroimaging has increased the perception of neurobiological underpinnings of PD and its various clinical presentations. Imaging of brain glucose metabolism, which may be precisely and consistently measured using ^18^F‐FDG PET, has been a significant contributor to this field. This method is largely utilized for normal clinical use. PDRP has correlations to cross‐sectional disease rigorousness and unified PD rating scale motor scores, and longitudinal disease progression and treatment response. However, it is observed that individuals having Parkinson's indications but no dopaminergic shortage do not exhibit PDRP, contrary to PD sufferers of dopaminergic scarcity. Additionally, the PDRP is not associated with tremors. The PDRP and group‐based metabolic patterns methods focus on regional ^18^F‐FDG uptake or quantitative properties of metabolically aberrant portions in PET images, without seeing the metabolic connections among regions. As a result, linked information of differences in metabolic topology may be lost.

The analysis of brain metabolic networks using graph theory has the potential to provide a personalized assessment of metabolic patterns that can predict disease conversion, leading to better insight of PD pathophysiology. This approach has identified both local and global metabolic abnormalities that are characteristic of PD and can serve as an effective diagnostic biomarker of earlier disease recognition. Herein, we introduce new strategy for constructing individual‐level metabolic brain networks in ^18^F‐FDG PET imaging that incorporates the concept of relative entropy. This approach preserves critical individual‐level information while enabling the testing of the JSSE method for predicting PD from NC, as assessed through clinical follow‐up.

As our JSSE brain connectome strategy may determine global and local graph metrics of metabolic network, it can detect PD's relevant properties. Our main finding was that normalized characteristic path length, assortativity coefficient, modularity score, and characteristic path length of PD patients were larger than of NC group, while synchronization and global efficiency were lesser in PD groups. When comparing the NC and PD groups, we observed changes in both the betweenness and degree exploration of the metabolic brain connectome. Our main finding was that PD is associated with modules or subnetworks disruption within global network, as well as damage of connectivity of these modules. These findings are in accordance to earlier research of metabolic networks, that have also shown similar network changes in PD.^26^, ^42^, ^46^

This also is revealed that motor networks explored under normal conditions are perturbed in PD. In this analysis from consensus important metabolic connections, prominent metabolic network was PUT–PCG pathway. The PUT is an important part of the BG, which is affected as a key node of the metabolic network and seems to be associated with impaired motor symptoms, which smartly theorized the details for bradykinesia and rigidity.^47^, ^48^ Beyond PUT–PCG pathway, THA–PCG and SMA were also shown to be involved. The SMA, a key region associated with motor symptoms, is also affected as a central node. These results can be assisted by experiments revealing differential involvement of BG and cortex–striatum–thalamus–cortex and that both structures can transform one another at subcortical level,^46^, ^49^ which is similar to classic group‐level methodology. The motor pathways and lateral prefrontal cortices receive projections from the dopaminergic system through caudate. The frontostriatal structure is closely linked to posterior parietal cortex and plays a role in executive and memory functions. It could also be seen from our results that the projection areas downstream of individual pathological connectivity in the ACG–PCL, DCG–PHG, and ACG pathways were significantly affected. Therefore, the akinesia‐rigidity‐related metabolic connectivity networks validated in our study might help understanding circuits' pathophysiology and explaining their specified functions in producing clinical indications. Besides these areas, our findings revealed that further connections (45 important connections) of PD sufferers were involved. This is vital to conduct more studies on bigger canvas and combining the clinical data and stratification. All abbreviations are given in Table S3.

As shown in our work, the brain's metabolic connectivity networks that may be affected by tremor symptoms associated with the cerebellum were not significantly affected. Previous studies have indicated that the PDRP is not linked to tremors,^15^, ^50^ possibly due to the differing pathophysiologic origins of bradykinesia/rigidity and tremors. Previous studies have identified tremor‐related regions that are the same as those given in “dimmer switch” theory.^51^ According to this theory, striato‐pallidal circuit initiates tremor episodes (light switch), while cerebello‐thalamo‐cortical circuit generates tremor and influences its amplitude (light dimmer).^45^, ^50^ In future, we will include the metabolic network related to the cerebellum in analysis, which may provide a better entry point for more clinical symptoms of PD, deeper mechanism research, and provide better diagnostic performance for PD diagnostic imaging markers.

Our brain connectome approach was able to effectively distinguish PD from NC by measuring both local network characteristics and entire system. Specifically, our main finding was that PD is characterized by a subnetworks or modules disruption within global network architecture, as well as losing connection of these modules. This is consistent with earlier PET experiments that show earlier collapse of brain modules linked with cognitive dysfunction onset. Our study demonstrates that metabolic connections can serve as effective biomarkers for identifying PD. Further, the topological information can also provide more discriminative information, since the multikernel support vector machine (MK‐SVM), which combines all of this information, provides a more accurate diagnostic result.

There are several limitations to our study that should be taken into account when reading the results. First, our datapoints were gathered retrospectively and only involved lesser patients. Moreover, we did not analyze other preoperative neuroimaging data or results from more advanced diagnostic tests such as structural MRI. Also, we implemented JSSE method for ^18^F‐FDG PET images without partial volume effect correction. In addition, our explanation of the link for PD pathology and metabolic network failure is unconfirmed by DAT neuroimaging and clinical manifestations. Therefore, future studies should include other types of data besides glucose metabolism to completely confirm metabolic connectome methodology's applicability.

## 4 CONCLUSIONS

In this study, we conducted an innovative connectome evaluation of ^18^F‐FDG PET images using unique JSSE entropy application, that was not used before for metabolic connectome analysis. Our approach provided new insights into the network abnormalities associated with PD. Further, our finding demonstrates that by combining outcomes from various ideas could provide exemplary methodology for identifying the PDs from NCs. As a naïve empirically validation, one limitation is that we only conduct the automated anatomical labeling (AAL) atlas for constructing brain network. In our future work, more atlas will be considered for some further multiscale study.

## 5 MATERIALS AND METHODS

### 5.1 Patients

Between January 2018 and December 2019, 49 patients were consecutively enrolled (33 males and 16 females, aged 53.94  ±  11.16 years) who were detected with idiopathic PD on the basis of International Parkinson and Movement Disorder Society diagnostic principles and underwent ^18^F‐FDG PET scans. Patients with a history of substance use disorder, psychiatric illness, intracranial operation, cerebral stroke, and head injury were excluded from the study. Complete clinical history about subjects is available in Table 3. Additionally, we recruited 49 NCs of same gender distribution, education, and age for obtaining normative data. No NCs had head injury, central nervous system disease, psychiatric illness, or cognitive impairment history. Every subject gave signed informed consent as per the Helsinki Declaration. The experiments were endorsed by Studies Institutional Review Board at Xiangya Hospital, Central South University.

**TABLE 3:** Clinical and demographic attributes regarding NCs and PD patients.

Variable	NC (*n* = 49)	PD (*n* = 49)	*p* Value
Age (years)	52.12 ± 9.84	53.94 ± 11.16	0.832
Sex (male/female)	30/19	33/16	0.577
Education (years)	13.44 ± 3.15	12.37 ± 4.06	0.163
Disease duration (months)	NA	60.2(13‐101)	NA
UPDRS‐III score	NA	23.2 ± 8.7	NA
Side of onset (left/right)	NA	23/26	NA
Levodopa response	NA	49 (100.0%)	NA
Rest tremor	NA	30 (61.2%)	NA
Fast clinical course	NA	13 (26.5%)	NA
Motor fluctuations	NA	35 (71.4%)	NA
Dyskinesias	NA	32 (65.3%)	NA
Dysautonomia	NA	31 (63.3%)	NA
Postural instability	NA	5 (10.2%)	NA
Mild cognitive impairment	NA	25 (51.0%)	NA
Dementia	NA	5 (10.2%)	NA
Anxiety/depression	NA	30 (61.2%)	NA
Hallucinations	NA	12 (24.5%)	NA
Psychosis	NA	6 (12.2%)	NA
Rapid eye movement sleep behavior disorder	NA	25 (51.0%)	NA
Family history	NA	12 (24.6%)	NA

NC, normal control; UPDRS, unified Parkinson's disease rating scale; PD, Parkinson's disease.

### 5.2 Acquisition of ^18^F‐FDG PET image and processing

The ^18^F‐FDG PET was accomplished at PET Center of Xiangya Hospital using a Discovery Elite PET/computed tomography (CT) scanner (GE Healthcare, Boston, MA, USA). To correct for photon attenuation, a CT transmission scan was conducted. Participants were administered with ^18^F‐FDG (3.7 MBq/kg) intravenously and instructed to rest in supine posture on PET scanner bed having closed eyes for 45−60 min. A 10‐min scan was then performed using a three‐dimensional (3D) model. The images were recreated using ordered‐subset expectation maximization algorithm with a 6‐iteration and 6‐subset method. The ^18^F‐FDG PET imaging data handling was done by employing the statistical parametric mapping (SPM) software (Wellcome Department of Cognitive Neurology, London, UK) applied on MATLAB. In this study, individual ^18^F‐FDG PET image volumes were normalized into standard stereotactic Montreal Neurological Institute space using nonlinear and linear 3D conversions. To compare every participant, images intensity was globally normalized. To segment cerebral cortex to 90 portions, with 45 regions for every hemisphere excluding cerebellum, AAL atlas was utilized.

### 5.3 Statistical analysis

All analyzes were performed on the Statistical Package for Social Sciences software (version 22). Differences in the demographic and clinical characteristics between the PD and NC groups were analyzed using Pearson's chi‐square test in cases of categorical variables, comparisons between groups in age and education were determined using the two‐sample *t*‐tests. The connectivity strength of the rich club, feeder and local connections between the PDs and NCs were compared using the two‐sample *t*‐test. *p* Values < 0.05 represented significant difference (*p* value was uncorrected).

### 5.4 Construction of individual JSSE metabolic network

The individual morphological brain networks are constructed by utilizing distribution‐divergence‐based methodology.^30^ In this study, we assume that ^18^F‐FDG PET signal in brain areas reflects metabolic connectivity that contribute to inter‐regional information transfer. A comparatively large resting S/N ^18^F‐FDG PET signal in region of interest (ROI) indicates glucose metabolic rate. Therefore, this linkage provides reasonable technique for analyzing inter‐neuronal information transfer. In previous studies, Kullback–Leibler (KL) divergence was utilized for measuring statistical association of resemblance of cerebral glucose metabolism between two regions^52^:

(1) $$D_{KL}\left(P||Q\right)=\int_{x}\left(P\left(x\right)\log\frac{P(x)}{Q(x)}\right)dx$$

where P and Q denote probability density functions (PDFs) of voxel intensities in ROIs pair. However, KL divergence is asymmetric. Therefore, in this study, we utilized JSSE for capturing statistical relation for cerebral glucose metabolism resemblance in any two portions, that can be used to set down individual metabolic connectivity. We denoted nodes of the brain by ninety regions of interest from AAL atlas parcellation for exhibiting individual metabolic networks. Voxels intensity in every ROI was gathered and employed for estimating PDF of relevant ROI by using kernel density estimation.^53^ We obtained metabolic connectivity as JS divergence as per following equation:

(2) $$D_{\mathrm{JS}}\;\;\left(P||Q\right)=\frac{1}{2}\;\left[D_{\mathrm{KL}}\left(P||M\right)+D_{\mathrm{KL}}\left(Q||M\right)\right]$$

where M=0.5×(P+Q) and $D_{\mathrm{KL}}\left(\cdot |\cdot \right)$ are KL divergence. Herein, we applied JS divergence as metabolic connectivity measure for constructing an adjacency matrix. By this, the adjacency matrix depicts pairwise metabolic connectivity, where strength of metabolic connection $W_{ij}\;$between ROI iand *j* may be indicated by relevant elements element$\;\mathrm{JSs}\;(P_{i}||P_{j})$ in adjacency matrix.

(3) $$W_{\mathrm{ij}}=\;\mathrm{JSs}\;\;\left(P_{i}\left|\right|P_{j}\right)=\exp\left(-D_{\mathrm{JS}}\;\left(P_{i}\left|\right|P_{j}\right)\right)\;$$

where $P_{i}$ and $P_{j}$ are the PDFs of ROI iand *j* estimated by kernel density estimation.

**Remark**: One advantage of the JSSE‐based individual metabolic brain network is that there is no negative connection network, due to the non‐negative of the JS divergence, which is more tolerant for the subsequent analysis.

### 5.5 Graph metrics computation

Our aim was to explore the changes in the reconfiguration design of individual brain metabolic connectomes in PD. Utilizing binary undirected matrices, we thoroughly evaluated local and global characteristics of functional brain system through Graph Theoretical Network Analysis Toolbox.^1^ Particularly, global metrics include modularity score (*Q*),^54^ global efficiency (*E*_global_), small‐world (σ), normalized characteristic path length (λ), clustering coefficient ($C_{p}$), normalized clustering coefficient (γ), and characteristic path length ($L_{p}$).^55^ In addition, the nodal properties analyzed in this study involved nodal clustering coefficient, shortest path length, betweenness centrality, nodal efficiency, and degree centrality. These quantities are defined in pipeline provided by Wang et al.^39^ Nodal and global graph metrics were utilized to study various connections designs in brain system. Especially, for ensuring network meets full connection and it is not too dense, we follow the standard pipeline provided by Gretna.^39^ Specifically, we sparsity network at various thresholds of sparsity (0.02–0.5, with 0.01 gradient), and total of 49 values of relevant node features in sparsity threshold was achieved. The total of 49 values were taken for every node (AUC) as input for classifier training. There was one value relating to one graph metric.

### 5.6 PD identification and feature combination

**FIGURE 4:**
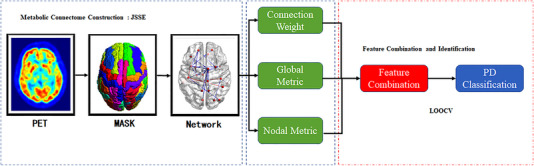
Classification procedures and data‐processing employed in our work. JSSE was adopted to create a metabolic network. The global and nodal graph metrics were calculated. Finally, MK‐SVM was implemented for combining the data for identifying PD.

Towards an accurate identification of the PDs from the NCs, we proposed to unite the data of connection weights, nodal, and global graph metrics. In this paper, we employed kernel combination strategy to combine data and utilized MK‐SVM for PD identification. To minimize hinderance from feature selection process, we used simple feature selection methodology (*t*‐test having *p* less than 0.05). Figure 4 illustrates all classification procedures and data‐processing used in this work. Details of MKSVM and feature selection are given in the Supplemental Materials.

## AUTHOR CONTRIBUTIONS

All authors contributed to the conception and design of this work. Material preparation, data collection, and analysis were performed by Weikai Li, Yongxiang Tang, Zhengxia Wang, Shuo Hu, and Xin Gao. The first draft of the manuscript was written by Weikai Li and Yongxiang Tang. Liling Peng, Shuo Hu, and Xin Gao commented on previous versions of the manuscript. All authors read and approved the final manuscript.

## CONFLICT OF INTEREST STATEMENT

The authors declare that they have no competing interests.

## ETHICS STATEMENT

The study protocol was approved by the Ethics Committee of the Xiangya Hospital Central South University (NO. 201412455). The procedures used in this study adhere to the tenets of the Declaration of Helsinki. Informed consent was obtained from all individual participants or legal guardians included in the study.

## Supporting information

Supporting Information

## Data Availability

The datasets and custom code utilized and/or generated during the current study can be obtained from the corresponding author upon reasonable request.

## Footnotes

1
www.nitrc.org/projects/gretna/
