# Supplementary material for: The reconfiguration pattern of individual brain metabolic connectome for Parkinson's disease identification
Source: MedComm (2020). 2023 Jun 27;4(4):e305. doi: 10.1002/mco2.305 (PMC10300308; doi:10.1002/mco2.305)
Supplement: Supplementary file 1 — Supporting Information [file MCO2-4-e305-s001.docx]

**Supplemental Materials for “****The Reconfiguration Pattern of Individual Brain Metabolic Connectome for Parkinson's Disease Identification”**

Weikai Li^1,2,3#^, PhD; Yongxiang Tang^2#^, MD, PhD; Liling Peng^3^, PhD, Zhengxia Wang^4^, PhD; Shuo Hu^2,5^*, MD, PhD; Xin Gao^3^, PhD*;

**Affiliations:**

^1^ College of Mathematics and Statistics, Chongqing Jiaotong University, Chongqing, P.R. China.

^2^ Department of Nuclear Medicine (PET Center), XiangYa Hospital, Changsha, Hunan, P.R. China.

^3^ Shanghai Universal Medical Imaging Diagnostic Center, Shanghai, P.R. China.

^4^ School of Computer Science and Cyberspace Security, Hainan University, Hainan, P. R. China.

^5^ Key Laboratory of Biological Nanotechnology of National Health Commission, Xiangya Hospital, Central South University, Changsha, Hunan, P.R. China.

**Multi Kernel Support Vector Machine**

The details of the MK-SVM method in this study can be conducted as follows. In particular, suppose that there are $n$ training samples with connections values and graph metrics, let $x_{i}^{1}$, $x_{i}^{2}$, and $x_{i}^{3}$ represent the connection weight, the graph metrics, and nodal graph metrics of the $i$-th sample, respectively. Denoting that $y_{i}\in\left\{ 1,-1 \right\}$ be the corresponding label; the MK-SVM solves the following primal problem:

|  | $\min_{W} \frac{1}{2}\sum_{m=1}^{3} \beta_{m}\left\Vert w^{m} \right\Vert^{2}+C\sum_{i=1}^{n} \xi_{i}$  $s.t. y_{i}(\sum_{m=1}^{3} \beta_{m}{(w^{m})}^{T}\phi^{m}{(x}_{i}^{m})+b)\geq1-\xi_{i}$  $\xi_{i}\geq0,i=1,2,...,n$ | (4) |
| --- | --- | --- |

where $\phi^{m}$ represents the transform from the original space in $m$-th data to the Represent Hilbert Kernel Space (RHKS), $w^{m}$ represents the hyperplane in RHKS, and $\beta_{m}$ denotes the corresponding combining weight on the $m$-th attribute. Next, the dual form of MK-SVM can be represented as:

|  | $\max_{\alpha} \sum_{i=1}^{n} \alpha_{i}-\frac{1}{2}\sum_{i,j} \alpha_{i}\alpha_{j}y_{i}y_{j}\sum_{m=1}^{3} \beta_{m}k^{m}(x_{i}^{m},x_{j}^{m})$  $s.t.\sum_{i=1}^{n} \alpha_{i}y_{i}=0$  $0\leq\alpha_{i}\leq C,i=1,2,\ldots n$ | (5) |
| --- | --- | --- |

where $k^{m}\left( x_{i}^{m},x_{j}^{m} \right)=\phi^{m}{(x_{i}^{m})}^{T}\phi^{m}{(x}_{j}^{m})$ and is the kernel matrix on the $m$-th data. After we trained the model, we tested the new samples $x=\left\{ x_{1},x_{2},\cdots,x_{M} \right\}$. The kernel between the new test sample and $i$-th training sample on the $m$-th modality is defined as $k^{m}\left( x_{i}^{m},x^{m} \right)=\phi^{m}\left( x_{i}^{m} \right)^{T}\phi^{m}(x^{m})$. In the end, the predictive level based on MK-SVM can be formulated as follows:

|  | $f\left( x_{1},x_{2},\ldots,x_{M} \right)=sign(\sum_{i=1}^{n} y_{i}\alpha_{i}\sum_{m=1}^{M} \beta_{m}k^{m}\left( x_{i}^{m},x^{m} \right)+b)$ | (6) |
| --- | --- | --- |

To illustrate the performance gain of the information combination from different views, such as connection and metrics, we employed the most commonly used and the simplest linear kernel as $k^{m}\left( x_{i}^{m},x_{j}^{m} \right)$, which is given as follows:

|  | $k^{m}\left( x_{i}^{m},x_{j}^{m} \right)={x_{i}^{m}}^{T}x_{j}^{m}$. | (7) |
| --- | --- | --- |

- 1. **Feature selection and validation**

To confirm the effectiveness of the proposed PD identification, we conducted the strictest nest leave-one-outcross-validation (LOOCV) strategy to verify the performance of the methods due to the small sample size, in which only one subject was left out for testing while the others are used to train the models and obtain the optimal parameters. For the choice of optimal parameters, an inner LOOCV was conducted on the training data using a grid-search strategy. The range of the hyper-parameter $C$ was $2^{-5}$to $2^{5}$ . Meanwhile, to alleviate the interference from the feature selection procedure, we selected the simplest feature selection method (t-test with p < 0.05) to select the nodal graph metric and the connection weight in our experiment.

**Classification Measurement**

The mathematical definitions of these 3 measures were given as follows:

|  | $Accuracy=\frac{TruePostive+TrueNegative}{TruePostive+FalsePostive+TrueNegative+FalseNegative}$, | (8) |
| --- | --- | --- |
|  | $Sensitivity=\frac{TruePostive}{TruePostive+FalseNegative}$, | (9) |
|  | $Specificity=\frac{TrueNegative}{TrueNegative+FalsePostive}$, | (10) |

*TruePositive* is the number of the positive participants that are correctly classified in the PD identification task. Similarly, *TrueNegative*, *FalsePostive* and *FalseNegative* are the numbers of their corresponding subjects, respectively. Also, the receiver operating characteristic curve (ROC) and AUC of these methods is also provided.

**Graph Metrics**

The definitions of the Graph Metrics are defined as follows:

Cluster coefficient $C_{p}$ measures the degree of clustering of a network and is another important parameter for measuring the network. It represents the possibility of neighbors of a node i being neighbors to each other. The value of cluster coefficient $C_{i}$ of node i is equal to the ratio of the number of actually connected edges ($e_{i}$) among the neighbors of the node to the maximum number of possible connected edges ($C_{i}=\frac{k_{i}\left( k_{i} - 1 \right)}{2}$), and $C_{p}$ is the average value of $C_{i}$

$$C_{p}=\frac{1}{N}\sum_{i}^{N} C_{i}$$

Since the cluster coefficient only considers the direct connections between neighbor nodes, the concept of local efficiency $E_{loc}$ was proposed. The local efficiency of any node $i$ is

$$E_{loc}\left( i \right)=\frac{1}{N_{G_{i}}\left( N_{G_{i}} \right)}\sum_{j\neq k} \frac{1}{l_{j,k}}$$

$l_{j,k}$ is the shortest path between node *j* and *k*. The local efficiency $E_{local}$ is the average value of each node:

$$E_{local}=\frac{1}{N}\sum_{i}^{N} E_{loc}\left( i \right)$$

The modularity ($Q$) of a brain network quantified the efficiency of segmenting a network into modules^1^. Modified greedy optimization algorithm was used as follows:

$$Q=\sum_{i=1}^{N_{m}} [{l_{i}}/L-{({d_{i}}/{2L})}^{2}]$$

where $N_{m}$represents the number of modules, $L$ is the total number of edges in the brain network, $l_{i}$is the number of within-module edges in the module $i$ and $d_{i}$ represents the sum of the linked edges at each node within the module $i$. In the present study, modified greedy optimization was applied to detect the modular structure

The shortest path ($L_{p}$) plays an important role in the information transmission of the network. It is a very important parameter to describe the internal structure of the network. The shortest path describes the optimal path for information from a node to another node on the network. Information can be transmitted faster through the shortest path, thus saving system resources.

$$L_{p}=\frac{1}{N\left( N-1 \right)}\sum_{j\neq k} l_{j,k}$$

Usually the shortest path length is calculated in a connected graph, because if there are disconnected nodes in the network, the shortest path length between the two nodes will be infinite. Therefore, global efficiency ($E_{global}$) was proposed

$$E_{global}=\frac{1}{N\left( N-1 \right)}\sum_{j\neq k} \frac{1}{l_{j,k}}$$

For more definition about graph metrics, please refer to ^2^

**Significant nodes of average degree in NC and PD groups.**

**The Significant nodes of average degree in NC and PD groups are given in Table S1**

**Table S1. 19 significant nodes of average degree in NC and PD groups.**

|  | NC | PD | **p-value** |
| --- | --- | --- | --- |
| **MFG.L** | 12.85745 | 13.95122 | 0.031289 |
| **ROL.L** | 11.44102 | 9.117347 | 0.002185 |
| **SMA.L** | 13.74214 | 15.23551 | 0.019944 |
| **OLF.L** | 1.741122 | 2.537143 | 0.011798 |
| **OLF.R** | 1.624388 | 2.171122 | 0.044952 |
| **ORBsupmed.L** | 9.476735 | 11.49398 | 0.02319 |
| **ORBsupmed.R** | 9.89449 | 11.73776 | 0.036806 |
| **ACG.R** | 7.774898 | 6.24102 | 0.016161 |
| **AMYG.L** | 0.02102 | 0.105306 | 0.003493 |
| **AMYG.R** | 0.025306 | 0.108571 | 0.004302 |
| **FFG.R** | 7.918469 | 6.584082 | 0.021963 |
| **PoCG.R** | 15.37347 | 14.055 | 0.026412 |
| **SMG.L** | 17.04286 | 15.35806 | 0.021154 |
| **THA.L** | 4.593061 | 6.773265 | 0.000653 |
| **THA.R** | 6.258469 | 8.104592 | 0.005116 |
| **HES.L** | 1.594082 | 2.565816 | 0.033751 |
| **STG.R** | 13.28173 | 14.81122 | 0.014103 |
| **MTG.L** | 18.08561 | 16.95786 | 0.012137 |
| **TPOmid.L** | 8.450306 | 6.572143 | 0.023848 |

NC, normal control; PD, Parkinson's disease

**Significant nodes of average betweenness in NC and PD groups.**

**The Significant nodes of average betweenness in NC and PD groups are given in Table S2**

**Table S2. 15 significant nodes of average betweenness in NC and PD groups.**

|  | NC | PD | **p-value** |
| --- | --- | --- | --- |
| **IFGtriang.R** | 1.165445 | 1.881727 | 0.049115 |
| **ROL.L** | 14.3233 | 7.91026 | 0.000901 |
| **SMA.R** | 29.50238 | 19.23082 | 0.004348 |
| **OLF.R** | 8.682077 | 15.84762 | 0.016143 |
| **SFGmed.R** | 23.48307 | 30.91199 | 0.009278 |
| **HIP.L** | 5.262653 | 10.74993 | 0.012116 |
| **SOG.L** | 24.10385 | 17.31138 | 0.023121 |
| **MTG.L** | 16.86597 | 12.40085 | 0.011204 |
| **SOG.R** | 20.12192 | 15.48497 | 0.048746 |
| **PCL.L** | 18.03614 | 12.75296 | 0.02918 |
| **CAU.R** | 13.97596 | 9.408317 | 0.046454 |
| **PUT.L** | 51.8123 | 37.28866 | 0.024638 |
| **THA.L** | 12.30414 | 28.51788 | 0.001588 |
| **STG.R** | 4.480052 | 7.879975 | 0.001181 |
| **TPOsup.R** | 4.366766 | 7.879856 | 0.041972 |

NC, normal control; PD, Parkinson's disease

**Abbreviations for ROIs**

The Abbreviations for ROIs are given in Table S3.

**Table S3. Abbreviations for ROIs**

| **Regions** | **abbr.** |
| --- | --- |
| Precental gyrus | PreCG.L |
| Precental gyrus | PreCG.R |
| Superior frontal gyrus, dorsolateral | SFGdor.L |
| Superior frontal gyrus, dorsolateral | SFGdor.R |
| Superior frontal gyrus, orbital part | ORBsup.L |
| Superior frontal gyrus, orbital part | ORBsup.R |
| Middle frontal gyrus | MFG.L |
| Middle frontal gyrus | MFG.R |
| Middle frontal gyrus, orbital part | ORBmid.L |
| Middle frontal gyrus, orbital part | ORBmid.R |
| Inferior frontal gyrus, opercular part | IFGoperc.L |
| Inferior frontal gyrus, opercular part | IFGoperc.R |
| Inferior frontal gyrus, triangular part | IFGtriang.L |
| Inferior frontal gyrus, triangular part | IFGtriang.R |
| Inferior frontal gyrus, orbital part | ORBinf.L |
| Inferior frontal gyrus, orbital part | ORBinf.R |
| Rolandic operculum | ROL.L |
| Rolandic operculum | ROL.R |
| Supplementary motor area | SMA.L |
| Supplementary motor area | SMA.R |
| Olfactory cortex | OLF.L |
| Olfactory cortex | OLF.R |
| Superior frontal gyrus, medial | SFGmed.L |
| Superior frontal gyrus, medial | SFGmed.R |
| Superior frontal gyrus, medial orbital | ORBsupmed.L |
| Superior frontal gyrus, medial orbital | ORBsupmed.R |
| Gyrus rectus | REC.L |
| Gyrus rectus | REC.R |
| Insula | INS.L |
| Insula | INS.R |
| Anterior cingulate and paracingulate gyri | ACG.L |
| Anterior cingulate and paracingulate gyri | ACG.R |
| Median cingulate and paracingulate gyri | DCG.L |
| Median cingulate and paracingulate gyri | DCG.R |
| Posterior cingulate gyrus | PCG.L |
| Posterior cingulate gyrus | PCG.R |
| Hippocampus | HIP.L |
| Hippocampus | HIP.R |
| Parahippocampal gyrus | PHG.L |
| Parahippocampal gyrus | PHG.R |
| Amygdala | AMYG.L |
| Amygdala | AMYG.R |
| Calcarine fissure and surrounding cortex | CAL.L |
| Calcarine fissure and surrounding cortex | CAL.R |
| Cuneus | CUN.L |
| Cuneus | CUN.R |
| Lingual gyrus | LING.L |
| Lingual gyrus | LING.R |
| Superior occipital gyrus | SOG.L |
| Superior occipital gyrus | SOG.R |
| Middle occipital gyrus | MOG.L |
| Middle occipital gyrus | MOG.R |
| Inferior occipital gyrus | IOG.L |
| Inferior occipital gyrus | IOG.R |
| Fusiform gyrus | FFG.L |
| Fusiform gyrus | FFG.R |
| Postcentral gyrus | PoCG.L |
| Postcentral gyrus | PoCG.R |
| Superior parietal gyrus | SPG.L |
| Superior parietal gyrus | SPG.R |
| Inferior parietal, but supramarginal and angular gyri | IPL.L |
| Inferior parietal, but supramarginal and angular gyri | IPL.R |
| Supramarginal gyrus | SMG.L |
| Supramarginal gyrus | SMG.R |
| Angular gyrus | ANG.L |
| Angular gyrus | ANG.R |
| Precuneus | PCUN.L |
| Precuneus | PCUN.R |
| Paracentral lobule | PCL.L |
| Paracentral lobule | PCL.R |
| Caudate nucleus | CAU.L |
| Caudate nucleus | CAU.R |
| Lenticular nucleus, putamen | PUT.L |
| Lenticular nucleus, putamen | PUT.R |
| Lenticular nucleus, pallidum | PAL.L |
| Lenticular nucleus, pallidum | PAL.R |
| Thalamus | THA.L |
| Thalamus | THA.R |
| Heschl gyrus | HES.L |
| Heschl gyrus | HES.R |
| Superior temporal gyrus | STG.L |
| Superior temporal gyrus | STG.R |
| Temporal pole: superior temporal gyrus | TPOsup.L |
| Temporal pole: superior temporal gyrus | TPOsup.R |
| Middle temporal gyrus | MTG.L |
| Middle temporal gyrus | MTG.R |
| Temporal pole: middle temporal gyrus | TPOmid.L |
| Temporal pole: middle temporal gyrus | TPOmid.R |

1. Newman, M. E., Finding community structure in networks using the eigenvectors of matrices. *Phys Rev E Stat Nonlin Soft Matter Phys* **2006,** *74* (3 Pt 2), 036104.

2. Boccaletti, S.; Latora, V.; Moreno, Y.; Chavez, M.; Hwang, D.-U., Complex networks: Structure and dynamics. *Physics reports* **2006,** *424* (4-5), 175-308.
